# Infection Patterns of *Albugo laibachii* and Effect on Host Survival and Reproduction in a Wild Population of *Arabidopsis thaliana*

**DOI:** 10.3390/plants14040568

**Published:** 2025-02-13

**Authors:** Ignacio Taguas, François Maclot, Nuria Montes, Israel Pagán, Aurora Fraile, Fernando García-Arenal

**Affiliations:** Centro de Biotecnología y Genómica de Plantas (CBGP UPM-CSIC/INIA), E.T.S.I. Agronómica, Alimentaria y de Biosistemas, Universidad Politécnica de Madrid UPM, Campus de Montegancedo, Pozuelo de Alarcón, 28223 Madrid, Spain; francois.maclot@inrae.fr (F.M.); nuria.montes.casado@gmail.com (N.M.); jesusisrael.pagan@upm.es (I.P.); aurora.fraile@upm.es (A.F.)

**Keywords:** white rust, Arabidopsis wild populations, coevolution, coinfection, virulence

## Abstract

*Albugo* spp. are biotrophic parasites that cause white rust in Brassicaceae species, with significant crop losses. The generalist *A. candida* and the specialist *A. laibachii* infect *Arabidopsis thaliana*, and the pathosystem Albugo–Arabidopsis is a model for research in molecular genetics of plant–pathogen interactions. The occurrence of infection by Albugo in wild populations of Arabidopsis and data on the genetics of resistance-susceptibility are compatible with a hypothesis of host–pathogen coevolution. However, the negative impact of Albugo infection on Arabidopsis—a requirement for coevolution—has not been shown under field conditions. To address this question, we analysed the demography and the dynamics of Albugo infection in a wild Arabidopsis population in central Spain and measured plant fitness-related traits. Infection increased mortality by 50%, although lifespan, the fraction of plants that reproduced and seed production were reduced only in plants from the spring cohorts. Despite these negative effects, simulations of demographic dynamics showed that the population growth rate remained unaffected even at unrealistically high infection incidences. The lack of negative effects in autumn–winter cohorts suggests compensatory mechanisms in longer-lived plants. Results support the hypothesis of Albugo–Arabidopsis coevolution.

## 1. Introduction

Pathogens may impose selective forces in their host plants, as infection may result in decreased host fitness, i.e., the reproductive success of the individual that is generally measured as the number of viable descendants [1,2,3,4]. In consequence, plants have developed a variety of defence mechanisms, and plants and pathogens may co-evolve [5]. Understanding plant–pathogen coevolution has received much attention from plant biologists, and research in this field has focussed on a few well-characterised systems, including the interaction of *Arabidopsis thaliana* (L.) Heynh. (from here on Arabidopsis) with species of the genus *Albugo*. The oomycete *Albugo candida* (Pers. Ex Hook) Kunze is a biotrophic parasite of plant species in the family Brassicaceae. The disease caused by *A. candida* is commonly known as white rust, as infection results in the production of erumpent, sub-epidermic, white sori that contain zoosporangia. White rust can cause important losses in brassica crops, including rapeseed (*Brassica napus* L.), turnip rape (*Brassica rapa* L.) and the different varieties of *B. oleracea* L. [6]. *A. candida* also infects a wide range of wild species in the Brassicaceae, being a generalist parasite with physiological races specialising in different host genera [6]. Isolates of different races cluster in different phylogenetic groups [7,8,9], and 17 lineages have been reported, each with a repertoire of effector candidate alleles that may explain host specificity [9]. Arabidopsis has been known to be a host of *A. candida* since early studies in the 1980s that allowed the establishment of this pathosystem as a model for research in molecular genetics and molecular ecology [10]. *A. candida* isolates of most races do not infect Arabidopsis [7,10,11], but isolates from Arabidopsis belong to Race 4, initially defined for isolates from *Capsella bursa-pastoris* L. [9]. The resistance of Arabidopsis to isolates of different races is due to a variety of hypersensitive resistance (HR) genes encoding nucleotide-binding domain (NB), leucine-rich repeat (LRR) immune receptors with different recognition specificities of *A. candida* isolates [7,11,12] that depend on the recognition of specific effectors [13]. Also, resistance to *A. candida* isolates from Arabidopsis has been reported in this host, and major NB-LRR genes mapped [14,15], but this resistance is found in less than 15% of 49 screened accessions from all over the world [16]. A second species that infects Arabidopsis *is Albugo laibachii*, Thines and Y.J. Choi, which, at odds with *A. candida*, is a specialist parasite of this host [17]. Resistance to *A. laibachii* also occurs in Arabidopsis, and, as for *A. candida*, it is not frequent, being found in about 14% or 10% of 126 screened accessions, depending on the challenging isolate [18].

Infection by *A. candida* has been reported in about 25–33% of monitored wild populations of Arabidopsis in England and the south of Scotland [16,19], which, together with data on the molecular genetics of resistance-susceptibility in the pathosystem, is compatible with a hypothesis of coevolution between Arabidopsis and *Albugo* sp. However, for host–parasite coevolution to occur, parasite infection must decrease host fitness, and host resistance must decrease parasite fitness, defined as its transmission success (number of new susceptible individuals colonized per infected individual) [20]. While the effects of HR on *Albugo* sp. fitness are obvious, to our knowledge it has not been shown that infection by *Albugo* sp. has a negative effect on the fitness of Arabidopsis under the conditions encountered in wild populations. This is the purpose of this study. We jointly analysed the demography of a wild Arabidopsis population in central Spain and the dynamics of *A. laibachii* infection, and in each monitored plant, we measured plant life history traits related to fitness components of survival and reproduction. Infection of Arabidopsis by *Albugo* sp. might also have indirect negative effects due to an increased susceptibility to infection by other pathogens, as infection of different hosts by *A. candida* results in the suppression of immunity to subsequent infections by a wide range of pathogens [9,21,22]. Over the years, infection by viruses, notably cucumber mosaic virus (CMV) and turnip mosaic virus (TuMV), was consistently high in the analysed Arabidopsis population [23]. Thus, we also analysed if infection by *Albugo* sp. was associated with infection by CMV and TuMV. Our data contribute to understand the epidemic dynamics of *A. laibachii* and allow to quantify the effects of infection on different components of the fitness of the host plant. Interestingly, the negative effects of *A. laibachii* infection on Arabidopsis survival and fecundity depended on the cohort to which each plant belonged. These results are significant, as they show that infection by *A. laibachii* may decrease the fitness of Arabidopsis plants in nature, which supports the hypothesis of Albugo–Arabidopsis coevolution.

## 2. Results

### 2.1. Characterization of the Albugo Isolates Infecting Arabidopsis in the Ciruelos de Coca Population

**Figure 1:**
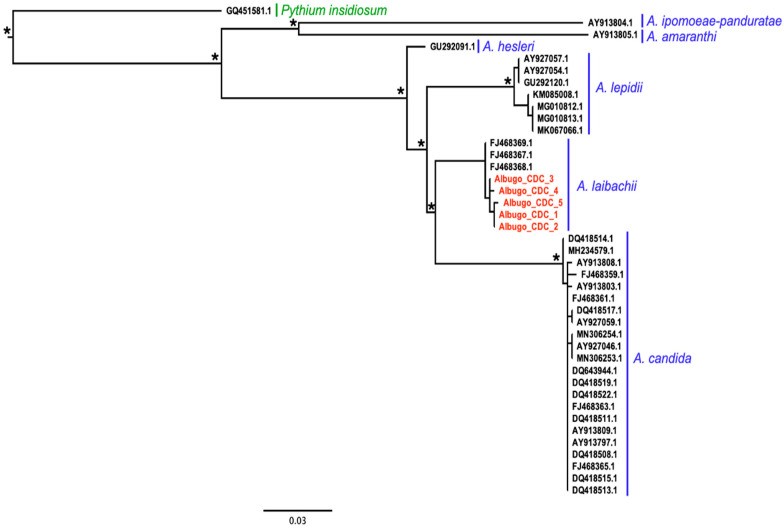
**Maximum-likelihood phylogeny of *Albugo* species based on the COX2 gene**. Isolates are identified by the GenBank accession number (see Table S1), except for those obtained in this work (in red) for which a three-letter code indicating the Arabidopsis population of origin (CDC: Ciruelos de Coca), and a correlative number, were used. The *Albugo* species represented in each monophyletic group are indicated in blue. Vertical blue lines delimit sequence clusters associated with a given *Albugo* species. The oomycete species used as an outgroup is indicated in green. Asterisks indicate nodes with bootstrap support higher than 80% based on 1000 replicates. Scale bar and branch lengths are in substitutions per site.

We obtained five COX2 sequences, each from a different infected plant (Figure S1). These sequences shared 99–100% nucleotide sequence identity. Phylogenetic analyses indicated that the COX2 sequences obtained from Arabidopsis plants collected in Ciruelos de Coca (Albugo_CDC_1 to 5, Figure 1) clustered within the monophyletic group formed by all the *Albugo* spp. sequences used to build the tree (Figure 1). Within this monophyletic group, Albugo_CDCs were phylogenetically closer to sequences from *A. laibachii* isolates (with 97–98% of nucleotide sequence identity) reported to infect Arabidopsis. The Albugo_CDC sequences clustered within the monophyletic group formed by species infecting members of the Brassicaeae, which did not include the other *Albugo* species used as reference (*A. amaranthi* and *A. ipomoeae-panduratae*) (Figure 1). Similar results were obtained using the ITS sequences (Figure S2).

### 2.2. Dynamics of Plant Demography and A. laibachii Infection

To analyse the demography of the Arabidopsis population, plants were considered as belonging to five age classes: recruits, plants at the rosette stage (with five or more rosette leaves), plants with flowers (with at least one flower), plants with siliques (with at least one silique) and dead plants. The total number of plants, and the number of plants at each stage at each visit, are shown in Table 1. Along the analysed period, a total of 259 recruits were counted, of which 243 (94%) developed rosettes of five or more leaves, 217 (84%) flowered, 188 (73%) developed siliques and produced seeds, and 104 (40%) had died at the date of the last visit. Most (90%) plants were born before 16 March, when the population reached a maximum, and decreased afterwards.

Table 1, and Figure S3, also show the dynamics of *A. laibachii* infection. Incidence reached 37.45% (97/259 plants). Incidence was low until the plant population growth decreased after 16 March, and then incidence grew until the end of April. Empirical data for the Disease Progress Curve (DPC) (Figure S3) could not be adjusted to any assayed model (exponential, Gompertz, logistic, log-logistic or Weibull), not even when only the growing portion of the curve was considered. During the period of incidence growth, the apparent infection rate was low, showing a maximum of 1.01 between 16 March and 30 March.

**Figure 2:**
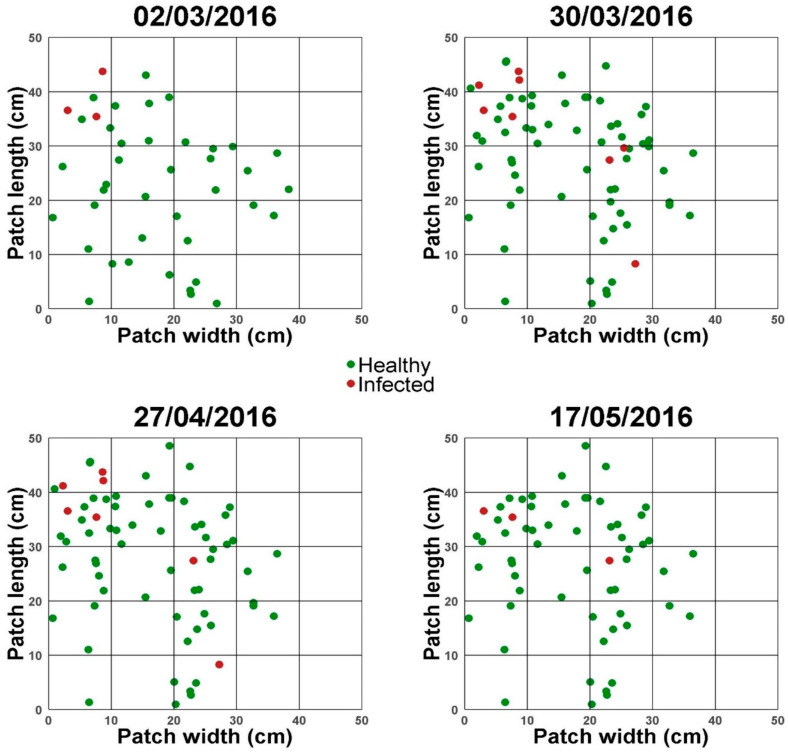
**Temporal change in the spatial distribution of infected and non-infected plants in Patch 1.** Position of plants in the grid cells is shown for four dates. Plants are shown as green (non-infected) or red (infected) circles.

Both plant density (24–348 plants/m^2^) and *A. laibachii* incidence (0–100%) varied largely among patches (Table S2), showing significant differences in a contingency test (G = 471.37, *p* < 0.0001, G = 825.59, *p* < 0.0001). The pattern of the spatial distribution of total plants and the infected ones at the population and patch spatial scales was analysed based on the data of Table S2 and on the basis of the location of plants in the different cells of the grids at each patch and visit (shown in Figure 2 for patch 1 and in Figure S4 for the rest of patches for the total number of plants), respectively. At the population scale, the dispersion index VM had a value of VM = 17.22 for all plants and of VM = 10.57 for infected plants, both values indicating an aggregated distribution. For each patch, VM values varied between 1.49 and 5.55 for total plants, and between 0.96 and 2.50 for infected plants. These results show that aggregation of total and infected plants increases with the spatial scale, being always lower for infected plants. A complementary analysis of spatial distribution at the patch scale was performed using SADIE. Results (Table S3) showed that total or infected plant aggregation was significant only for patch 1 and, marginally, for infected plants in patch 5. The aggregation maps for total and infected plants for patch 1, and their association (Figure S5) showed a single aggregation focus for both plant types.

### 2.3. Effect of A. laibachii Infection on Arabidopsis Fitness

The effect of infection by *A. laibachii* on two plant fitness components, survival and fecundity, was analysed. Survival was estimated from the fraction of plants alive at the end of the studied period. Fecundity was estimated from the fraction of plants that reproduced, i.e., produced mature siliques, and from the summation of the length of all siliques produced per plant, silique length (SL) and seed number per silique (SN) being positively correlated (SN = 2.38 + 28.44 SL, R = 0.87, *p* < 0.0001).

At the time of the last visit (7 June) 104 plants were dead, of which 39 plants (47.12%) had been infected. Of the 155 plants still alive, 48 (30.97%) were infected, showing that the fraction of infected plants that had died is higher than expected by chance ($\chi_{1}^{2}$ = 6.26, *p* = 0.0124), a result compatible with a negative effect of *A. laibachii* infection on Arabidopsis survival. The lifespan of the 104 plants dead by 7 June is known, which is not the case for the 155 survivors. To estimate the lifespan of the survivors, we assumed all of them were dead by June 14, which is a reasonable guess according to our monitoring of this Arabidopsis population since 2005 [23]. The lifespan of infected plants was significantly longer than the lifespan of non-infected plants both when only the 104 dead plants were analysed (68.94 ± 4.25 days vs. 51.47 ± 4.30 days; W_1_ = 1773, *p* = 0.0055) or when all the 259 plants were analysed (90.20 ± 3.08 days vs. 81.98 ± 2.50 days, W_1_ = 9554, *p* = 0.0032). A generalised linear model (GLM) with lifespan as a response variable, considering the patch as a random factor and infection status as a fixed factor showed that lifespan did not depend on patch, infection status or the interaction between both factors either for the set of 104 dead plants (F_6,92_ = 4.2500, *p* = 0.18 for patch, F_1,92_ = 0.80, *p* = 0.4431 for infection status, F_4,92_ = 1.00, *p* = 0.4112 for the interaction) or for the total set of 259 plants (F_6,247_ = 6.32, *p* = 0.0894 for patch, F_1,247_ = 3.40, *p* = 0.4763, F_4,247_ = 0.75, *p* = 0.5594 for the interaction). As the patch was not a factor on lifespan, a GLM was used with infection status as the sole fixed factor, which showed that the lifespan was longer for infected plants (F_1,102_ = 7.03, *p* = 0.0087 for the set of 104 dead plants; F_1,257_ = 8.12, *p* = 0.0053 for the set of 259 total plants).

As Table 1 shows, plants belonged to different cohorts, defined according to the date they were first identified (recruits). Lifespan depended on the cohort, and, thus, the effect of infection on lifespan was analysed separately for the different cohorts. The lifespan of infected and non-infected plants of the cohorts of plants first identified on 21 February or 2 March did not differ: 97.7 ± 3.13 days vs. 99.7 days ± 3.53 days for infected and non-infected plants in the 21 February cohort, W_1_ = 1899, *p* = 0.4233; 94.78 ± 5.74 days vs. 83.39 ± 4.75 days in the 2 March cohort W_1_ = 207, *p* = 0.598. However, infection reduced the lifespan of plants first identified on 16 March (60.42 ± 6.93 vs. 75.97 ± 4.34 days; W_1_ = 121, *p* = 0.005) or later (32.25 ± 10.9 vs. 51.26 ± 4.54 days) but this last difference was only marginal (W_1_ = 20.5, *p* = 0.082) due to the low number of plants in the last cohort (Table 1). The effect of infection on lifespan thus is higher the shorter the lifespan, which is negatively correlated with the date plants were first identified (R = −0.52, *p* < 0.0121, Table S4). Because of the dynamics of infection, most infected plants, 72 out of the total of 97 (74.23%) belonged to the first cohort of 21 February, while only 49 of the 162 plants that were never infected (30.25%) were identified at that date. This explains that when all plants are analysed together, infection is associated with a longer lifespan despite its association with a higher mortality. In agreement with this conclusion, when for the 72 infected plants of the first cohort, the relationship between the date of infection and lifespan was analysed, it was found that both variables were positively correlated (R = 0.26, *p* = 0.0280), indicating that the earlier the infection after plant birth the shorter the lifespan.

Regarding the effect of *A. laibachii* infection on plant fecundity, 34/97 (35.1%) infected plants and 37/162 (22.8%) non-infected plants failed to produce siliques, showing that the probability of reaching the reproductive stage was lower for infected plants ($\chi_{1}^{2}$ = 3.95, *p* = 0.0468). The mean value of the length of all siliques (total silique length) produced per plant did not differ for infected and non-infected plants (6.79 ± 9.19 vs. 6.81 ± 8.56 cm, W_1_ = 3967, *p* = 0.9332). A GLM in which patch was a random factor and infection status a fixed factor showed that total silique length did not depend on patch (F_6,176_ = 0.80, *p* = 0,6791) or infection status (F_1,176_ = 0.26, *p* = 0.7142), but depended on the interaction between both factors (F_4,176_ = 3.283, *p* = 0.0132), the effect of infection on silique length varying among patches. The cohort also had an effect on the impact of infection on total silique length, which was the same for infected and non-infected plants of the cohorts of 21 February (7.7 ± 9.15 vs. 8.13 ± 8.62, W = 1138, *p* = 0.4399) or 16 March (2.43 ± 1.89 vs. 3.39 ± 2.34 cm, W = 56, *p* = 0.5102) but shorter for infected plants of the 2 March cohort (2.20 ± 1.52 vs. 9.18 ± 12.99 cm, W = 194, *p* = 0.0400). Thus, *A. laibachii* infection had a negative effect on fecundity by reducing the probability of plants reaching the reproductive stage, and, among plants that reproduced, seed production was decreased depending on the site (patch) and plant cohort.

### 2.4. Association Between A. laibachii and Virus Infection

Infection by cucumber mosaic virus (CMV) and turnip mosaic virus (TuMV) was also monitored in the Arabidopsis population (Tables S5 and S6). Of the 97 plants infected by *A. laibachii*, 36 were also infected by CMV, while of the 162 plants not infected by *A. laibachii*, 46 were infected by CMV. These results show no association between infection by both parasites in a contingency test ($\chi_{1}^{2}$ = 1.75, *p* = 0.1862). No association was found between infection by *A. laibachii* and TuMV ($\chi_{1}^{2}$ = 0.29, *p* = 0.5894) either. Accumulation of CMV did not differ between Arabidopsis plants infected or not by *A. laibachii* (2.66 × 10^−5^ ± 3.73 × 10^−5^ vs. 3.96 × 10^−5^ ± 4.50 × 10^−5^ ng/µg of total RNA; W_1_ = 983, *p* = 0.3040). Accumulation of TuMV did not differ between *A. laibachii* infected and non-infected plants (7.26 × 10^−5^ ± 2.36 × 10^−5^ vs. 2.74 × 10^−5^ ± 2.65 × 10^−5^ ng/µg of total RNA; W_1_ = 238, *p* = 0.2537).

### 2.5. Effect of Infection by A. laibachii on the Dynamics of the Arabidopsis Population

**Figure 3:**
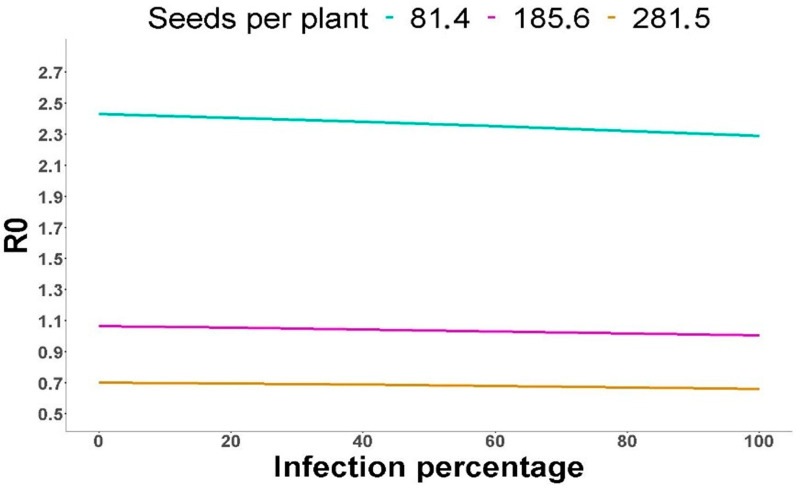
**Variation in the value of the basic reproduction number, R0, as a function of the incidence of *Albugo* sp. infection.** R0 values are shown for three different values of the number of seeds produced per plant.

To explore the effect of *A. laibachii* infection on the dynamics of the Arabidopsis population, we used the data from the previous sections in models of state transition matrices. We used state transition matrices with the age states that have been defined above (i.e., recruits, plants at the rosette stage, plants with flowers and plants with siliques) and the demographic values of Table 1, except for the transition between seeds produced in the previous season and recruits, as we did not have data on seed production per plant in 2015. We had data on seed production per plant in 2012, 2013 and those here estimated for 2016, which all showed a large variation. Thus, matrices were run using the minimum mean number of seeds produced per plant (81.4 seeds/plant, data of 2012), the maximum value (281.5 seeds/plant, data of 2013) and the mean of the three values (185.6), and each of these three values matrices were run considering *A. laibachii* incidences of 0, 10, 20, 30, … 100%. In Tables S7 and S8, we show, as examples, the matrices for the mean value of seed production and incidences of 0 and 100%. Figure 3 shows the variation in the Arabidopsis basic reproduction number with *A. laibachii* incidence for three values of seed production per plant. In all cases, the effect of infection on the dynamics of the host population was very small or negligible. For each state transition matrix, a sensitivity matrix was computed (Tables S9 and S10), which showed that the parameter with the highest influence on the dynamics of the population is the fecundity parameter, i.e., the number of seeds produced per plant. The number of seeds produced per plant relative to the number of recruits the following season is very high, which results in a low impact of infection in the host population dynamics.

## 3. Discussion

In this work, we test the hypothesis that infection by *A. laibachii* has a negative impact on the fitness of Arabidopsis under natural field conditions, a prerequisite [20] for the Albugo–Arabidopsis coevolution assumed from what is known on the genetics of the interaction [7,9,10,11,12]. For this purpose, we jointly monitored the dynamics of *A. laibachii* infection and the plant population demography in a wild Arabidopsis population located at Ciruelos de Coca, in central Spain [23]. According to the COX2 and ITS sequences, the specimens collected at Ciruelos de Coca belong to the species *A. laibachii*, previously reported to infect Arabidopsis. In the analysed Arabidopsis population, as is the case for Iberian populations from a variety of habitats, plants flower in spring and belong to different cohorts that either germinate in the autumn and overwinter as rosettes, or germinate in the spring [24,25]. The Ciruelos de Coca Arabidopsis population has been monitored for virus infection since 2005 [23], and data on infection by other pathogens or herbivory were also collected. Infection by *A. laibachii* was not detected before 2013, and in later years, infected plants were detected in a varying fraction of the surveyed patches of the population, between 25 and 66% depending on the year. This is a frequency of occurrence by site similar to that reported in UK populations, where incidence was not estimated [16,19]. At the CDC, incidence was low, below 5%, prior to 2016. In 2016, more than one-third of the plants were infected, and this high incidence allowed us to analyse the patterns of infection and to compare the life-history traits of a number of infected and non-infected plants sufficient to estimate the effects of infection on the host plant fitness.

Our data indicate that *A. laibachii* infection in autumn would explain at least a fraction of the infections detected at the first visit, 21 February, and infection occurs again in spring, in April (Table 1). This is in agreement with optimal conditions of 10–15 °C for zoospore release reported for *A. candida* [26], as mean temperature and mean maximum temperature were below these values after November and before late March (https://servicio.mapa.gob.es/websiar/SeleccionParametrosMap.aspx?dst=1 (accessed on 12 February 2025)). During periods of favourable conditions, few cycles of asexual reproduction seem to occur, as suggested by low values of the apparent infection rate. Meteorological conditions, however, do not explain the high incidence in 2016 compared with previous years, as neither temperature, nor rainfall, nor relative humidity differed significantly among years https://servicio.mapa.gob.es/websiar/SeleccionParametrosMap.aspx?dst=1 (accessed on 12 February 2025)). It could be that primary inoculum was introduced in the Arabidopsis population around 2013, and then it built up in subsequent years, and/or that prior to 2013, *A. laibachii* occurred as a vertically transmitted endophyte, as has been reported for *Albugo* sp. in wild Brassicaceae [27,28]. The factors that determine the transition to symptom development, production of sporangia and horizontal transmission are unknown [28]. Interestingly, the distribution of infected plants was less aggregated than that of total plants, and aggregation was detected at the population scale rather than at the patch scale. This suggests that the scale of inoculum dispersal is lower than the distance between patches, in the order of a few meters. Thus, horizontal transmission could occur among plants with different genotypes, as plants with the same genotype would be highly aggregated around the site of the parent plant.

Infection by *A. laibachii* resulted in a high increase, of about 50%, in mortality compared with non-infected plants. This result is in contradiction with an apparent increase in plant lifespan associated with infection. However, the longer lifespan of infected plants is an artefact due to the high proportion of plants from autumn/winter cohorts, which have a longer lifespan than those of spring cohorts, regardless of infection status. When the effect of infection was analysed considering the cohort to which the plants belonged, it was found that infection reduced the lifespan of plants from the spring cohorts, but not those from the autumn/winter cohorts. The effect of *A. laibachii* infection on plant survival translates into a direct effect on the probability of plants reaching the reproductive stage, which is decreased by about 54%. In addition, infection also may affect the fecundity of plants that reached the reproductive stage, but this effect was only significant for plants of the cohort of 2 March and not for those of earlier or later cohorts. Thus, the infection of Arabidopsis by *A. laibachii* has a negative impact on two major components of plant fitness, survival and fecundity, as has been reported for infection by *A. candida* in a wild population of *C. bursa-pastoris* [29]. An important finding of our study is that the effects of infection on the survival and fecundity of Arabidopsis depend on the cohort to which the plant belongs. The effect of the cohort on plant defence against herbivores has been analysed in field (common garden) and greenhouse experiments (e.g., [30,31,32]) but, to our knowledge, not in the wild cohorts with no human intervention, which determine actual plant demography in the field. Also, we are not aware of any study on the effect of cohort in plant responses to pathogens. Thus, we can only hypothesize on the nature of the cohort-dependent compensatory mechanism observed. The reduced or null effect of infection on the plants from the longer-lived cohorts cannot be attributed to a larger size of plants in the autumn cohorts, as both the number of rosette leaves (6.44 ± 0.17 and 6.20 ± 0.16 for the autumn and spring cohorts, respectively) and rosette diameter (1.07 ± 0.04 and 0.91 ± 0.03 cm, for the autumn and spring cohort, respectively) were similar for plants of both cohorts at 30 March, when infection rates of *A. laibachii* were highest. Later in spring, plants of the spring cohorts grew faster than those of the autumn cohorts, so that by 27 April, the rosette diameters were 0.96 ± 0.05 cm for plants in the autumn cohorts and 1.32 ± 0.08 cm for those in the spring cohorts. These data strongly suggest compensatory mechanisms based on resource re-allocation from growth to survival and reproduction, which would be more effective in the longer-lived and slow-growing plants of the autumn/winter cohorts, resulting in a higher tolerance to infection, defining tolerance as the ability of the host to limit the damage caused by a given parasite burden [33]. A similar compensatory mechanism has been reported in relation to infection by CMV [34] and in a variety of plant–pathogen interactions [33].

In addition to the direct effects of *A. laibachii* infection on Arabidopsis survival and fecundity, indirect effects may derive from an increased susceptibility to infection by other pathogens. It has been reported that infection of different Brassicaceae hosts, including Arabidopsis, by *A. candida* results in the suppression of immunity to subsequent infections by incompatible races of this oomycete or a wide range of other pathogens, such as *Hyaloperonospora parasitica* or *Erysiphe* spp. [21,22]. Infection of Arabidopsis by *A. candida* is also associated with a broader spectrum of infecting parasites and with higher titres of parasites as varied as chytridiomycetes, fungi, bacteria and rickettsiales [9]. Our previous observations over the years, showed that infection by *Hyaloperonospora* or fungi was even less frequent than infection by *A. laibachii* in the studied Arabidopsis population, but the incidence of virus infection was consistently high [23]. Thus, we analysed if infection by *A. laibachii* was associated with infection with the two viruses most prevalent in 2016, CMV and TuMV. No association between *A. laibachii* and CMV or TuMV infection was found, nor the titres of these viruses differed between plants infected or not infected by *A. laibachii*, suggesting that *A. laibachii* suppression of host defences may not affect virus infection, a topic that deserves further study.

Despite the negative effects of *A. laibachii* infection on Arabidopsis plants, simulations of demographic dynamics under a wide range of conditions showed that effects on the value of the basic reproduction number, which indicates population growth rate, are very low, even at unrealistically high incidence. Sensitivity analyses showed that population growth depends primarily on the fecundity parameter, with a very high ratio of seeds produced to recruits, that is not much diminished by infection. Similarly, the incidence of up to 30% of *A. candida* in a wild metapopulation of *Erysimum menziesii* (Hook.) Wettst., another semelparous brassicacea, caused high mortality but did not control population size because of the very high seed production of survivors [35]. In both pathosystems, however, infection by *Albugo* sp. may shape the genetic structure of the population by selecting against the more susceptible genotypes. It is worth mentioning that the Ciruelos de Coca Arabidopsis population is genetically heterogeneous [25], although susceptibility of the different genotypes to *A. laibachii* has not been tested.

In summary, this study demonstrates for the first time that *Albugo* sp. infection may have a significant negative effect on the fitness of Arabidopsis plants under natural field conditions, which may select for resistance and result in plant–pathogen coevolution. However, one may speculate that because high variation in incidence over seasons, as shown for the studied population, the selection for resistance may not be strong, which agrees with the relatively low frequency of resistant accessions [16,18]. Another important result is that the negative effects of *A. laibachii* infection depended on the cohort to which the plant belonged. This result underscores that the effects of disease on the fitness of Arabidopsis plants in the field, and possibly of other annual plants as well, cannot be analysed without considering the adscription of plants to different cohorts, as otherwise, data may lead to spurious conclusions.

## 4. Materials and Methods

### 4.1. Field Sample and Data Collection

**Figure 4:**
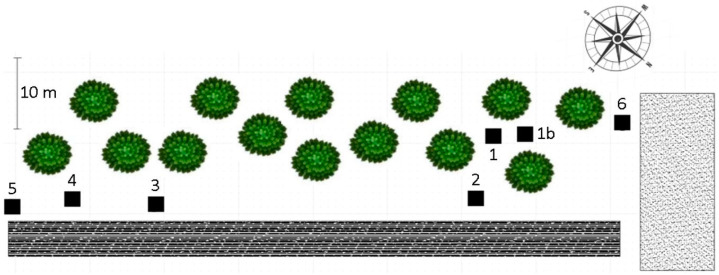
**Spatial location of the sampled patches within the Ciruelos de Coca *Arabidopsis thaliana* wild population**. Black squares and numbers (1, 1b to 6) correspond to the location of the patches analysed in this study. Green circles represent the location of trees in the sampled area. Black square indicates concrete road and grey square represents a sand path.

The studied population of Arabidopsis is located in Ciruelos de Coca, northern central Spain (41°12′ N, 4°31′ W), at 789 m altitude in a pine (*Pinus pinaster* Arr.) wood (Figure S1) on siliceous sands under low anthropic disturbance [23]. During the period of this study, the mean temperature at the site varied between 4.7 °C in February and 18.8 °C in June, and rainfall was 173.4 mm. Between 15 March and 30 April, when most Albugo infections occurred, the mean temperature was 7.67 °C, the mean maximal temperature was 14.1 °C, and rainfall was 79.8 mm (meteorological data accessed at https://servicio.mapa.gob.es/websiar/SeleccionParametrosMap.aspx?dst=1 (accessed on 12 February 2025)). Arabidopsis plants were aggregated in patches over an ~1275 m^2^ area, and seven patches along an ~90 m transect (Figure 4) were visited 9 times between 21 February and 7 June 2016, in the dates shown in Table 1. A 50 × 50 cm grid, divided into 25 10 × 10 cm cells, was positioned in each patch, and at each visit, plants growing within the grid were monitored. Each plant growing inside each cell of the grid was tagged, its position in the grid mapped, and for each plant and visit, data were collected on the following variables related to plant life history: the number of rosette leaves, rosette diameter, number of flowers, number of siliques and silique length. Only siliques of a length of ≥5 mm were counted, as shorter siliques did not produce seeds. All measurements were recorded in cm. The dates of the appearance of new plants, plants with flowers, plants with siliques and plant death were also recorded. In the last visit of April, one silique was collected from each of 20 randomly sampled plants, and silique length and number of seeds per silique were measured to determine the relationship between silique length and number of seeds per silique. At each visit, infection by *Albugo* sp. was also recorded. Plants were rated as infected based on the occurrence of white blisters (sori) in leaves (Figure S1 and Table S11). Infection severity was not rated, as blisters only occurred in rosette leaves, and affected on average 3 (1–9) leaves of rosettes that on average had 7 (4–10) leaves.

When the rosette diameter was ≥1 cm or the rosette had 6 or more leaves, a 3 mm diameter disk was collected for the detection of virus infection. Leaf disks were placed in 1.5 mL Eppendorf tubes and kept at 4 °C during transportation to the laboratory, where they were frozen in liquid nitrogen and stored at −80 °C. To control for any possible effect of tissue collection on plant life history, during the visit on 16 March, a set of 41 plants growing out of the monitored grids was tagged. Leaf disks were excised from odd-numbered plants and data on lifespan and total silique length per plant were compared for odd (clipped) and even (non-clipped) numbered plants. Neither lifespan (*Z* = 0.834, *p* = 0.361) nor total silique length per plant (*Z* = 0.901, *p* = 0.342) differed between clipped and non-clipped plants, indicating that taking samples of leaf tissue did not affect these life-history traits.

### 4.2. Detection of Virus Infection

Infection by cucumber mosaic virus (CMV) or turnip mosaic virus (TuMV) was detected using quantitative reverse transcription and polymerase chain reaction (RT-qPCR). RNA was extracted from leaf disks of field plant samples using the Trizol method with the NZYol reagent (NZYtech, Lisbon, Portugal) following the supplier’s recommendations. Primers for RT-qPCR of CMV were 5′-CGTTGCCGCTATCTCTGCTAT-3′(Fwd) and 5′-GGATGCTGCATACTGACAAACC-3′ (Rev), which amplify a 70 bp fragment of the coat protein gene of the Fny_CMV (positions 1661–1731 in Acc. No. D10538.1). Primers for UK1-TuMV were 5′-TGTTCGGCTTGGATGGAA-3′ (Fwd) and 5′-TTAACGTCCTCGGTCGTAT-3′ (Rev), amplifying a 70 bp fragment of the coat protein gene of the virus (positions 9515–9585 in Acc. No. NC_004422.1). RT-qPCR was performed in duplicate for each RNA extract of each plant sample. Each assay was performed in a volume of 8 μL of mix, i.e., 5 μL of SYBR Green (Agilent, (Santa Clara, USA), 0.2 μL of Reverse primers (Sigma Aldrich, Saint Louis, USA), 0.2 μL of Forward primers (Sigma Aldrich), 0.1 μL of DTT (Agilent), 0.5 μL of Reverse Transcriptase-DNA Polymerase (Agilent) and 2 μL of autoclaved water. Reverse transcription was at 50 °C, followed by 5 s denaturation at 95 °C and 40 cycles of denaturation at 95 °C and amplification at 60 °C. For quantification, data from field samples were compared with a known curve of 10× dilutions of an initial concentration of 100 ng/µL of purified RNA of either Fny-CMV or UK1-TuMV, diluted in RNA extracts of non-inoculated Arabidopsis plants grown in the greenhouse, and these extracts were also used as negative controls (data for each sample are shown in Table S11).

### 4.3. Temporal and Spatial Analyses of Infection

The temporal dynamics of infection by *Albugo* sp. were analysed by adjusting the empirical data of the disease progress curve (DPC) to different growth models with regression analyses using function *drm* in the *drc* R package as in [36]. The spatial distribution of infected plants at the population and patch grid scale was analysed with the dispersion index VM, estimated as the rate of the variance to the mean of the number of elements present in each studied unit, where VM values >1 indicate aggregation of elements [37]. The spatial distribution of infected plants within each patch grid was also analysed using SADIE (Spatial Analysis by Distance IndicEs) [38], which measures the total effort (in terms of distance moved) the individuals in the observed sample must expend to move to extreme arrangements, in which the individuals in the samples are either spaced as uniformly (regularly) or are as aggregated (crowded) as possible. On the basis of the number of elements in each grid cell, SADIE computes the following indices: D, measuring the minimum distance that sample individuals must move to generate a distribution with the same number of elements per cell, which is compared with values generated from random distributions of samples to yield the aggregation index, Ig. Ig from the sample is compared with values generated from random distributions among sample units yielding a probability *p* of the null hypothesis of a uniform distribution. SADIE allows us to compare aggregation in two associated populations through the comparison of D for each population and each space point. SADIE results were visualised with SURFER9 [39].

### 4.4. Characterization of the Albugo Isolate Infecting Arabidopsis in the Ciruelos de Coca Population

Characterization of the *Albugo* specimens infecting plants in the Ciruelos de Coca Arabidopsis population was based on the partial sequence of the cytochrome *c* oxidase subunit II (COX2) region of mitochondrial DNA obtained from infected plants. For that, DNA was extracted from 0.1 g of Arabidopsis rosette leaves showing *Albugo* blisters by homogenizing the tissue in EB buffer (2% CTAB, 1.4M NaCl, 20 mM EDTA and 100 mM Tris) and further separation of nucleic acids using chloroform [40]. For the amplification of the partial COX2 gene, forward (5′-GGCAAATGGGTTTTCAAGATCC-3′) and reverse (5′-CCATGATTAATACCACAAATTTCACTAG-3′) primers, yielding an expected fragment of 567 nt and designed by [41], were employed. PCRs were conducted in 25 μL reaction volumes, with each reaction tube containing 3 μL of template DNA solution (approximately 100 ng), 5 μL of 5x buffer (50 mM KCl, 100 mM, Tris–HCl, pH 8.0, 0.1% Triton X-100 and 15 mM MgCl_2_), 3 μL of 2.25 mM dNTP, 1 μL (each) of 100 M primers, 0.4 μL Taq polymerase (5 U/μL) and 7.35 μL ddH_2_O. The thermal cycling parameters were the following: denaturation for 30 s at 96 °C, annealing for 30 s at 57 °C and extension for 1 min at 72 °C. Thirty-five cycles were performed with both the first denaturation and last extension times extended to 4 min. The success of the amplification was monitored with electrophoresis on 0.8% agarose gel. Amplicons of the expected size were sequenced using the primers above. Phylogenetic relationships between the obtained sequences (Acc. N. PV092575-PV092579) and those from other species of the genus *Albugo* were studied by comparing it with publicly available partial COX2 sequences from 35 *Albugo* isolates (Table S1). We included in the analysis sequences from six different *Albugo* species: *A. candida*, *A. laibachii*, *A. lepidii*, *A. amaranthi*, *A. hesleri* and *A. ipomoeae-panduratae*. For the first three species, several sequences per species were included as these were the ones with which the sequences of our isolates had higher nucleotide sequence homology. The other three species, with only one representative sequence per species, were included for reference. Also, a sequence of *Pythium insidiosum*, the oomycete phylogenetically closest to *Albugo* [42], was used as an outgroup. Sequences were aligned using Muscle5 [43]. Utilizing this alignment, a Maximum-likelihood (ML) tree was inferred using IQtree 2.2.2.6 [44] and incorporating TPM2u+F+R2 as the best-fitted nucleotide substitution model, as determined by ModelFinder [45]. Node support was obtained using ultrafast bootstrap with 1000 replicates [46]. Using the same DNA extracts, we also amplified the ITS region of rDNA with forward DC6 (5′-GAGGGACTTTTGGGTAATCA-3′) and reverse L0 (5′-GCTTAAGTTCAGCGGGT-3′) primers, amplification products being of a length between 1200 and 1350 bp and including a partial 18 S and complete ITSregion (ITS1, 5.8 S rDNA and ITS2), as designed by [8]. The sequence was limited to the partial 18 S and full-length ITS1 (Acc. No. and PV072565, PV072566, PV072567, PV072557 and PV072558). Following the same procedure as for COX2 sequences, a phylogenetic tree was constructed using the JC+G4 as determined by ModelFinder. In this case, only *A. leibachii*, *A. lepidii* and *A. candida* sequences were included, as no equivalent sequences for the other *Albugo* species were publicly available. For the same reason, *Pythium volutum* was used as an outgroup instead of *P. insidiosum*.

### 4.5. Statistical Analyses

The independence of variables in contingency tables was analysed by means of a *χ*^2^ test or, if entry values in tables were below 5, by a G test. The normality of variable distributions was analysed using the Shapiro–Wilk test of residuals. As no variable was normally distributed, mean values were compared using the Mann–Whitney non-parametric test implemented in R package (version 4.1.2). The Pearson correlation between variables, which is robust to distributions departing from normality, was implemented in the same programme. The effect of environmental factors (infection status and/or spatial location) on the plant life history variables was analysed using generalised linear models (GLM) implemented in SPSS Statistics 21 SPSS 17.0 (SPSS Inc., Chicago, IL, USA) after determining the distribution (gamma) that better fitted the data using R *fitdistr* function from the *fitdistrplus* library (version 1.2.2). All the statistical methods above are described in [47]. Demographic parameters were estimated from state transition matrix projection models that relate the number of individuals in any particular state category at time t to that at time *t* + 1 [48], implemented in the *Pop Tools* extension of Excel. For demographic analyses, the numbers of seeds produced by plants in the years 2012 (81.4 seeds/plant) and 2013 (281.5 seeds/plant), determined from direct counts of 20 plants randomly collected in the population, were used in addition to the data of 2016.

## 5. Conclusions

This study demonstrates for the first time that *Albugo* sp. infection may decrease the survival and reproduction of Arabidopsis plants under natural field conditions and that the negative impact of infection on the host plant fitness may select for resistance. Thus, plant–oomycete interaction has reciprocal negative effects for both the host (pathogen virulence) and the pathogen (host resistance), a condition for host–pathogen coevolution. Consequently, the results of this study support the hypothesis of Albugo–Arabidopsis coevolution, which had been assumed on the bases of molecular genetic analyses of this plant–pathogen interaction. The negative effects of infection depended on the cohort to which the plant belonged, which underscores the need to consider the host demography in the analyses of the effects of disease on the fitness of host plants in the field.

## Figures and Tables

**Table 1:** Temporal evolution of *A. thaliana* demography and infection by *Albugo* sp.

Date	Recruits ^1^	New Plants with 5 Rosette Leaves ^2^	New Plants with Flowers ^3^	New Plants with Siliques ^4^	Dead Plants ^5^	Total Plants ^6^	Number of New Infections	Total Number of Infections	Incidence (%)
21 February	121	83	0	0	0	121	29	29	23.97
2 March	60	26	0	0	2	179	2	29	16.2
16 March	51	45	0	0	6	224	5	33	14.73
30 March	12	46	39	1	15	221	32	64	28.96
14 April	8	33	94	78	13	217	11	68	31.34
27 April	7	10	45	85	11	213	17	78	36.62
9 May	0	0	30	20	14	199	1	74	37.19
17 May	0	0	9	3	18	181	0	63	34.8
7 June	0	0	0	1	26	155	0	48	30.97
**Total**	**259**	**243**	**217**	**188**	**104**	**259**	**97**	**97**	**37.45**

^1^ Plants first identified on the indicated date. ^2^ Plants that had reached a rosette size of ≥5 leaves on the indicated date. ^3^ Plants that were first identified with at least 1 flower on the indicated date. ^4^ Plants that were first identified with at least 1silique of ≥5 mm long on the indicated date. ^5^ Plants that were first identified as dead on the indicated date. ^6^ The number of total plants includes those that had reached a rosette of ≥5 leaves, or that had flowered or set fruit at earlier dates and, thus, is not the summation of the data in columns to the left (columns 2–5) minus the number of death plants.

## Data Availability

The original contributions presented in this study are included in the article/Supplementary Materials; further inquiries can be directed to the corresponding author/s.

## Supplementary Materials

The following supporting information can be downloaded at https://www.mdpi.com/article/10.3390/plants14040568/s1, Figure S1: Habitat of the Ciruelos de Coca *Arabidopsis thaliana* population (left), patch of plants within a quadrat (centre) and sori of *Albugo laibachii* in the adaxial side of rosette leaves (right); Figure S2: Maximum-likelihood phylogeny of *Albugo* species based on the ITS sequence; Figure S3: Temporal variation in the number of infected and non-infected plants; Figure S4: Spatial distribution of total plants for each patch; Figure S5: Spatial distribution of plants infected and not infected by *Albugo* sp.; Table S1: *Albugo* and oomycete sequences used for phylogenetic analyses; Table S2: Total number of plants and number of plants infected by *A. laibachii* per patch; Table S3: Analysis of the spatial distribution of total and infected plants on each patch; Table S4: Life expectancy as a function of cohort; Table S5: Coinfection *A. laibachii*—CMV; Table S6: Coinfection *A. laibachii*—TuMV; Table S7: Transition matrix for 185.6 seeds/plant, 0% incidence; Table S8: Transition matrix for 185.6 seeds/plant, 100% incidence; Table S9: Sensitivity analysis for the transition matrix for 185.6 seeds/plant, 0% incidence; Table S10: Sensitivity analysis for the transition matrix for 185.6 seeds/plant, 100% incidence; Table S11: Raw data obtained in this work.
