# Supplementary material for: Infection Patterns of Albugo laibachii and Effect on Host Survival and Reproduction in a Wild Population of Arabidopsis thaliana
Source: Plants (Basel). 2025 Feb 13;14(4):568. doi: 10.3390/plants14040568 (PMC11859388; doi:10.3390/plants14040568)
Supplement: Supplementary file 1 [file plants-14-00568-s001.zip › Supplementary Figures.pdf]

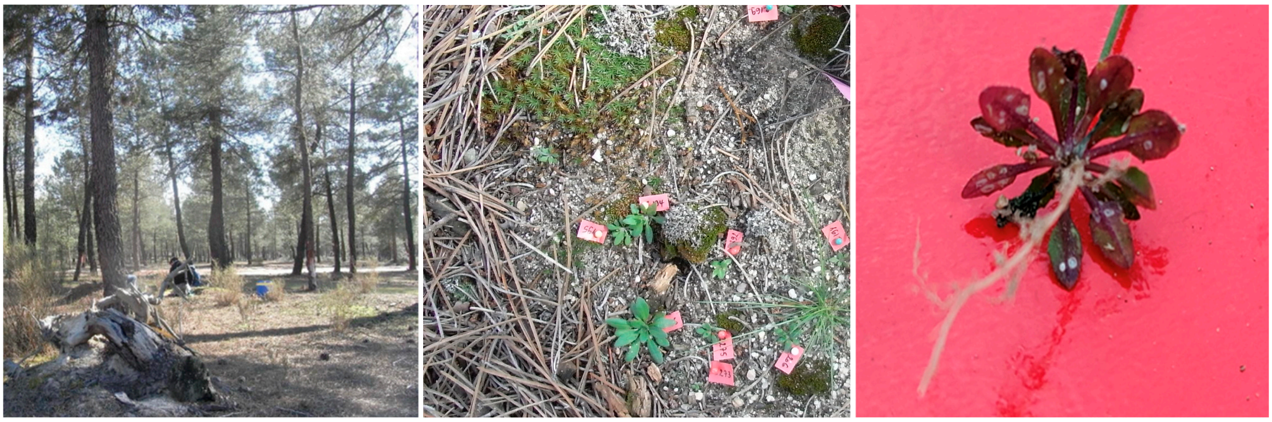

Figure S1. Habitat of the Ciruelos de Coca *Arabidopsis thaliana* population (left), patch of plants within a quadrat (center) and sori of *Albugo laibachii* in the adaxial side oof rosette leaves (right).

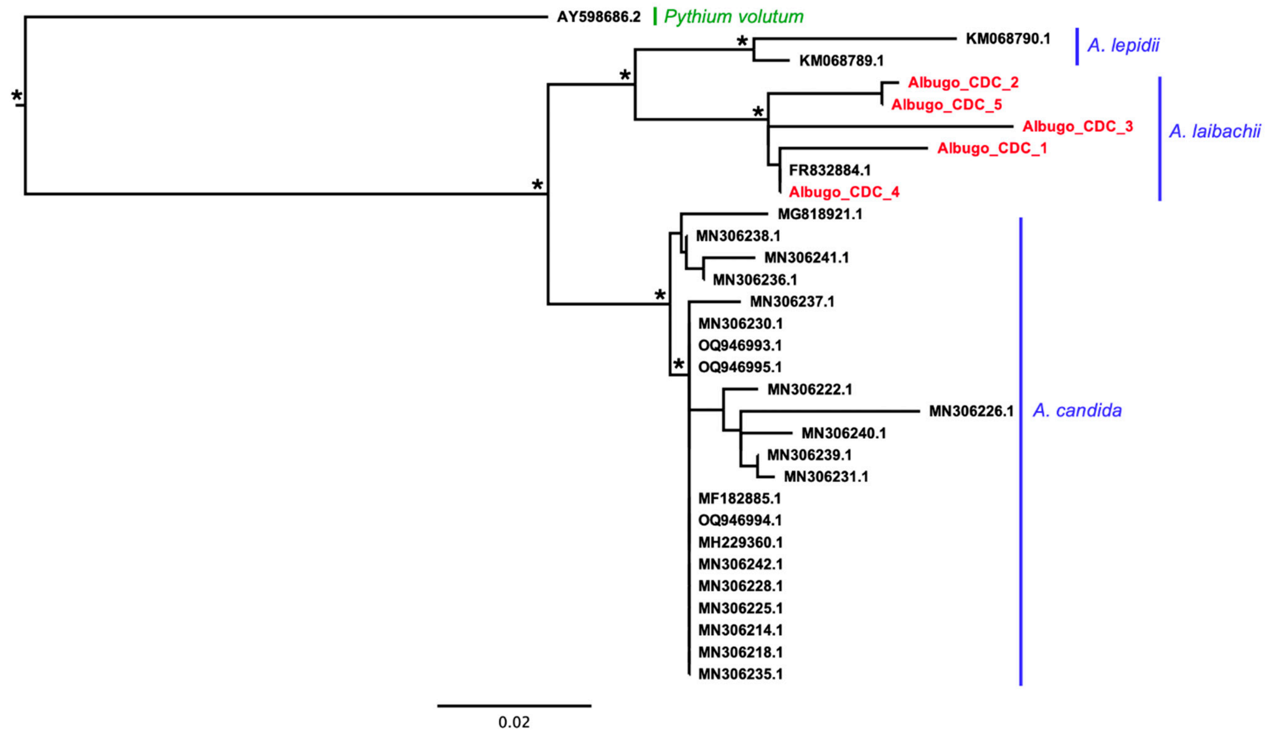

Figure S2. Maximum-likelihood phylogeny of *Albugo* species based on the ITS sequence. Isolates are identified by the GenBank accession number (see Table S1), except for those obtained in this work (in red) for which a three-letter code indicating the Arabidopsis population of origin (CDC: Ciruelos de Coca), and a correlative number, were used. The *Albugo* species represented in each monophyletic group are indicated in blue. Vertical blue lines delimit sequence clusters associated with a given *Albugo* species. The oomycete species used as outgroup is indicated in green. Asterisks indicate nodes with a bootstrap support higher than 80% based on 1000 replicates. Scale bar and branch lengths are in substitutions per site.

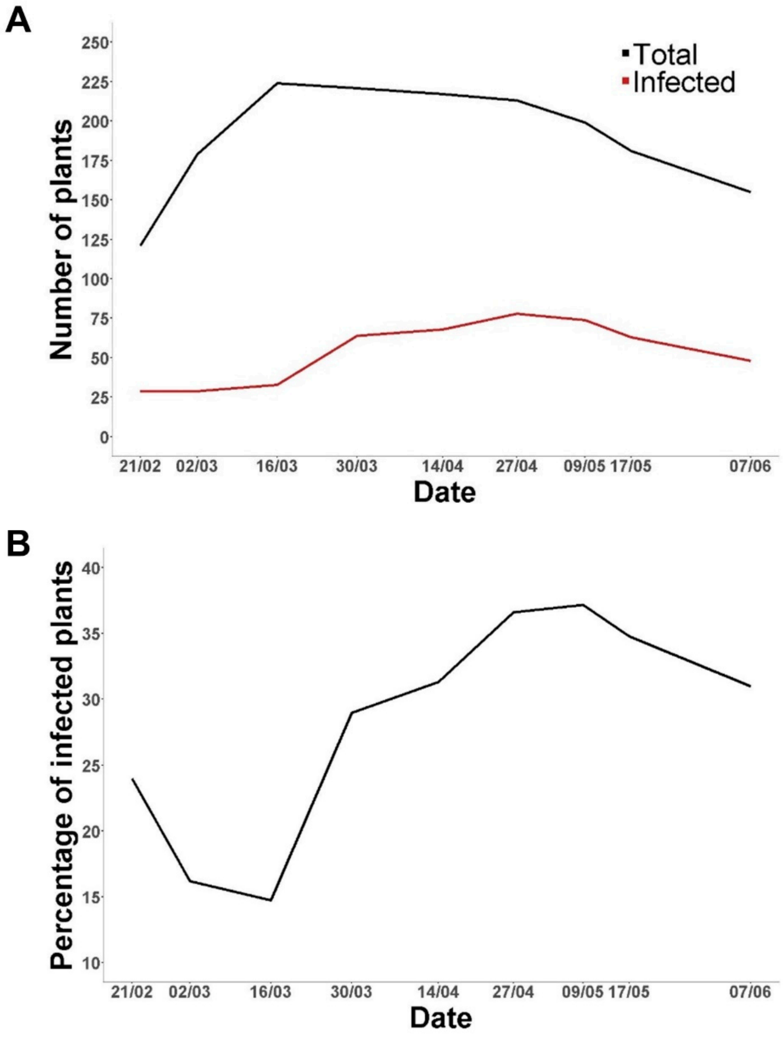

Figure S3. Temporal variation in the number of infected and non-infected plants. (A) shows the total number of total and infected plants (black and red lines) at each visit. **(B)** shows percentage of infection at each date.

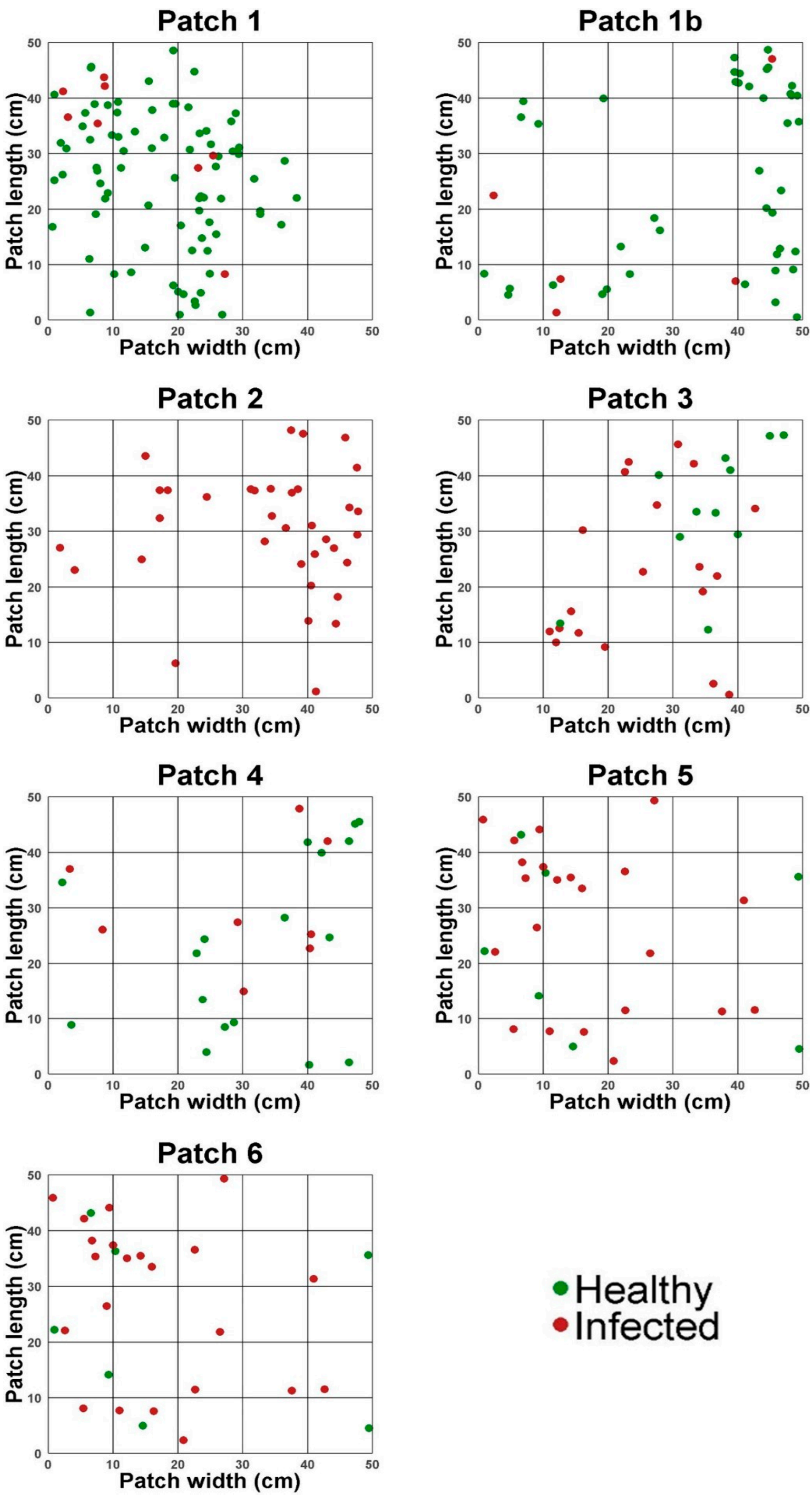

Figure S4. Spatial distribution of total plants for each patch. Position of all plants, aggregated over survey dates, in the grid cells is shown for the seven analysed patches. Plants are shown as green (non-infected) or red (infected) circles.

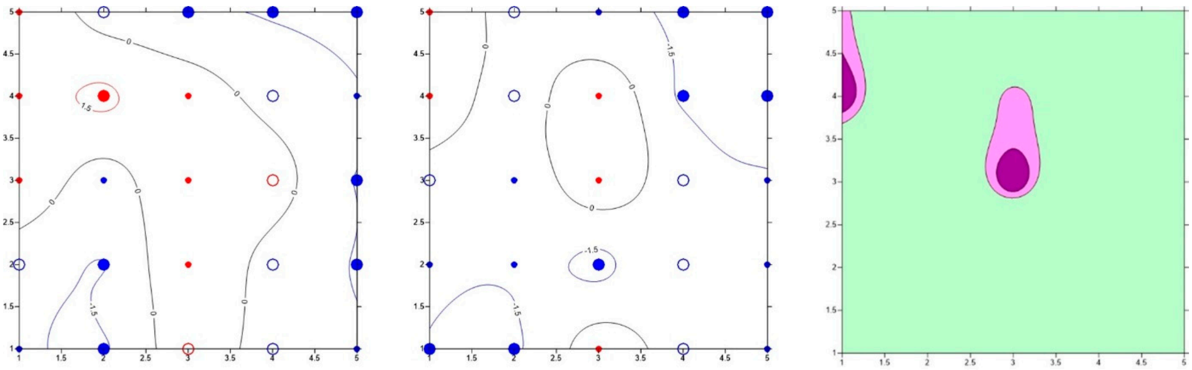

$I_g = 1.32$ $P_g = 0.0444$

$I_g = 1.33$ $P_g = 0.0365$

$I_g = 0.67$ $P_g = 0.0004$

Figure S5. Spatial distribution of plants infected and not infected by *Albugo* sp. The left panel shows the aggregation map of *A. thaliana*, the middle panel shows the aggregation map of *Albugo* sp., and the right panel shows the association map of both. In the aggregation maps, red dots indicate foci and blue dots indicate gaps in the distribution of total and infected plants, respectively, in a theoretical regular grid, and contour lines delimit these foci and gaps. In the association map, dark purple indicates significant association. I_g and P_g indicate aggregation/association index and aggregation/association probability.
